# Stay on Track: A Pilot Randomized Control Trial on the Feasibility of a Diet and Exercise Intervention in Patients with Breast Cancer Receiving Radiotherapy

**DOI:** 10.1158/2767-9764.CRC-23-0148

**Published:** 2024-05-07

**Authors:** Gopika SenthilKumar, Aronne M. Schottstaedt, Lindsay L. Peterson, Lauren N. Pedersen, Christopher R. Chitambar, Alexis Vistocky, Anjishnu Banerjee, John M. Longo, Tracy Kelly, Adam Currey, Melinda R. Stolley, Carmen Bergom

**Affiliations:** 1 Department of Physiology and Anesthesiology, Medical College of Wisconsin, Milwaukee, Wisconsin.; 2 Department of Radiation Oncology, Medical College of Wisconsin, Milwaukee, Wisconsin.; 3 Division of Medical Oncology, Department of Medicine, Washington University School of Medicine, St. Louis, Missouri.; 4 Alvin J. Siteman Cancer Center, Washington University in St. Louis, St. Louis, Missouri.; 5 Department of Radiation Oncology, Washington University School of Medicine, St. Louis, Missouri.; 6 Division of Medical Oncology, Department of Medicine, Medical College of Wisconsin, Milwaukee, Wisconsin.; 7 Division of Biostatistics, Medical College of Wisconsin, Milwaukee, Wisconsin.; 8 Division of Hematology and Oncology, Medical College of Wisconsin, Milwaukee, Wisconsin.

## Abstract

**Purpose:**

Among patients with breast cancer undergoing radiotherapy, posttreatment cardiovascular disease and worsened quality of life (QoL) are leading causes of morbidity and mortality. To overcome these negative radiotherapy effects, this prospective, randomized clinical trial pilots a 12-week Stay on Track exercise and diet intervention for overweight patients with nonmetastatic breast cancer undergoing whole-breast radiotherapy.

**Experimental Design:**

The intervention group (*n* = 22) participated in three personal exercise and dietary counseling sessions, and received three text reminders/week to adhere to recommendations. The control group (*n* = 22) was administered a diet/exercise information binder. All patients received a Fitbit, and at baseline, 3 months, and 6 months, measurements of biomarkers, dual-energy X-ray absorptiometry scans, QoL and physical activity surveys, and food frequency questionnaires were obtained. A satisfaction survey was administered at 3 months.

**Results:**

Stay on Track was well received, with high rates of adherence and satisfaction. The intervention group showed an increase in self-reported physical activity and preserved QoL, a decrease in body mass index and visceral fat, and higher American Cancer Society/American Institute of Cancer Research dietary adherence. The control participants had reduced QoL, anti-inflammatory markers, and increased metabolic syndrome markers. Both groups had decreased overall body mass. These changes were within group effects. When comparing the intervention and control groups over time, there were notable improvements in dietary adherence in the intervention group.

**Conclusions:**

Targeted lifestyle interventions during radiotherapy are feasible and could decrease cardiovascular comorbidities in patients with breast cancer. Larger-scale implementation with longer follow-up can better determine interventions that influence cardiometabolic health and QoL.

**Significance:**

This pilot study examines cardiometabolic benefits of a combined diet and exercise intervention for patients with breast cancer undergoing radiotherapy. The intervention included an activity tracker (FitBit) and text message reminders to promote adherence to lifestyle interventions. Large-scale implementation of such programs may improve cardiometabolic outcomes and overall QoL among patients with breast cancer.

## Introduction

The addition of adjuvant radiotherapy to early-stage breast cancer confers benefits in local control and survival. However, radiotherapy can negatively impact patients’ quality of life (QoL) and the risk of subsequent cardiovascular disease (CVD; refs. 1, 2). Breast cancer therapy, including chemotherapy and radiotherapy, is also associated with a decrease in lean mass and an increase in adipose tissue (3, 4). Adiposity/obesity further increases the risk of cancer-related mortality (5), and increases levels of inflammatory cytokines (6), contributes to leptin and adiponectin dysregulation (7, 8), and increases insulin resistance (9). Aberrations in adiponectin and leptin levels (too high or too low) can also contribute to and predict adverse cardiovascular events (10–12), and chronic inflammation is a known driver of CVD (13). As such, the cardiometabolic changes associated with breast cancer therapy can potentiate and worsen CVD.

Lifestyle modifications in physical activity and dietary intake are attractive solutions to improve both QoL during cancer treatment and posttreatment CVD risk. A recent meta-analysis of outcomes from randomized controlled trials of exercise interventions for patients with breast cancer between 2000 and 2018 reported that exercise interventions (usually 150 minutes per week of moderate to vigorous exercise) have a positive impact on QoL as well as CVD risk factors such as body fat, body mass index (BMI), and physical fitness (14). Lifestyle interventions can also prevent negative changes in body composition (15) and improve metabolic biomarkers and insulin resistance in overweight patients with breast cancer (16). Weight reductions, which are often associated with lifestyle interventions, can help normalize resting metabolic rates (RMR). RMR is commonly elevated in overweight patients and contributes to a positive energy balance that promotes chronic weight gain (17). Furthermore, regular exercise has been shown to lower the serum concentrations of proinflammatory cytokines in patients with breast cancer (18). Despite these benefits, few randomized trials of exercise interventions during radiotherapy for breast cancer have been published (19–21).

In addition to exercise, studies suggest that a healthy diet can decrease the risk of breast cancer recurrence (22), mitigate fatigue (23), and potentially improve CVD risk (24) in patients with breast cancer by promoting beneficial changes in cardiometabolic markers and body weight (25, 26). However, the association between diet and QoL is poorly understood. A recent systematic review identified only one trial that exclusively studied the effect of dietary intervention on QoL in patients with breast cancer (27), and only a few studies have assessed the impact of a combined diet and exercise intervention on QoL in patients with breast cancer undergoing adjuvant therapy (26, 28, 29).

The goal of this randomized clinical trial was to pilot a 12-week Stay on Track (SOT) exercise and diet intervention for patients with nonmetastatic breast cancer and a BMI greater than or equal to 25 undergoing whole-breast radiotherapy. The unique aspects of SOT include (i) integration of Fitbit for motivation/self-monitoring, (ii) focus on the effects of American Cancer Society (ACS) guidelines on QoL and cardiometabolic markers, (iii) implementation in radiation oncology, (iv) focus on women during radiotherapy rather than after completion of adjuvant therapies (30), and (v) a cognitive-behavioral approach grounded in social cognitive theory (SCT) to encourage self-motivated changes. The primary aim of this study was to establish the feasibility of SOT and provide preliminary data on changes in QoL, body composition, inflammatory and metabolic CVD biomarkers, and nutrition to support a fully powered randomized controlled trial to examine its efficacy.

## Materials and Methods

### Recruitment and Eligibility

The patients were screened by phone or in-person by study coordinators between March 2017 and August 2018. Inclusion and exclusion criteria are listed in Supplementary Table S1, with the representativeness of study participants described in Supplementary Table S2. Briefly, eligible patients had nonmetastatic breast cancer and were going to receive whole-breast radiotherapy, with a BMI ≥25 or not meeting ACS guidelines for physical activity and diet (31). Full Institutional Review Board approval was obtained from the Medical College of Wisconsin and all participants provided written informed consent. The study was conducted in accordance with the Belmont Report and the U.S. Common Rule.

### Procedure

**FIGURE 1:**
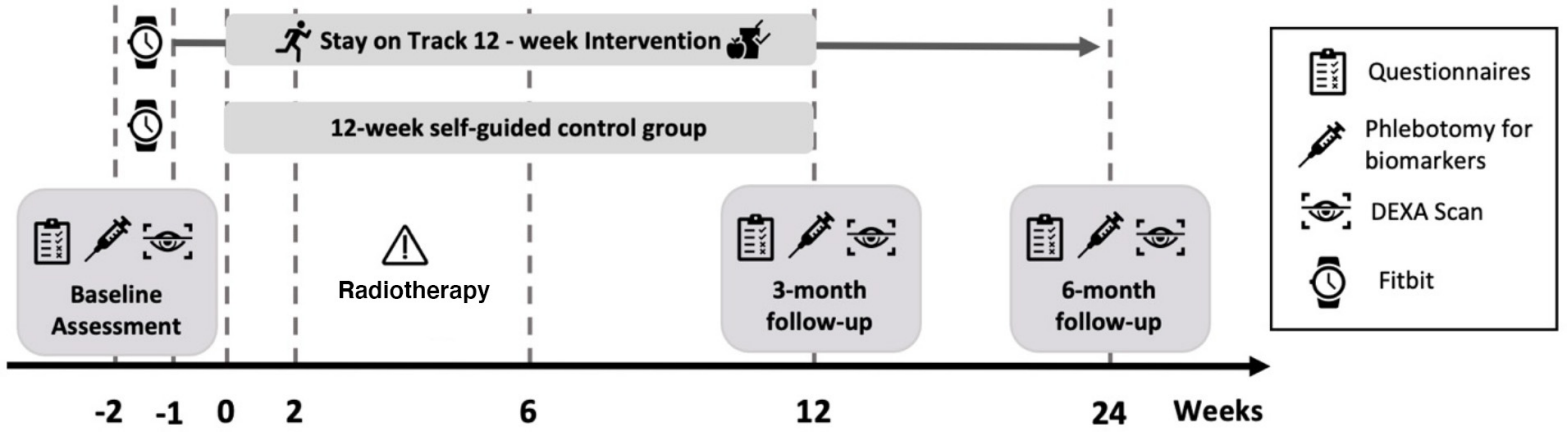
Study timeline.

This pilot study was a prospective phase II randomized controlled trial. Following informed consent, a baseline assessment was scheduled prior to the start of radiotherapy (∼2-week window). The 60-minute assessment included questionnaires, height/weight, dual-energy X-ray absorptiometry (DEXA) scan, self-reported list of comorbidities, and phlebotomy for biomarkers (study measures detailed in Supplementary Table S1). Participants were instructed to fast for at least 8 hours prior to blood draw. All participants were given a Fitbit to wear for 1 week to establish the baseline activity level. Participants were then randomly assigned to the SOT group or 12-week self-guided control group using the OnCore clinical trial management system (study timeline detailed in Fig. 1). Randomization was stratified by factors that can affect treatment side effects: menopausal status (premenopausal/postmenopausal) and BMI of 25 or above, and randomization was conducted after the baseline assessment to minimize the influence of arm assignment on baseline levels of activity. The control group was asked to stop using the Fitbit after the baseline week to avoid confounding motivation but were allowed to keep their Fitbit. They received binders containing information on activity and diet (similar to those received by the intervention group) at 24 weeks postintervention. The intervention participants received daily reminders during the 1-week baseline data collection to remind them of wearing the Fitbit. Throughout the duration of SOT, these texts also encouraged adherence to ACS guidelines. This text messaging strategy has been successfully used in previous lifestyle interventions (32). Both groups completed 3-month and 6-month follow-up visits, identical to their baseline assessments, with fasting for 8 hours prior to each visit. A satisfaction questionnaire was administered at 3 months.

Encrypted aggregate activity data for the intervention group were downloaded from Fitabase. The percentage of patients who wore the device per day was calculated. Patients were deemed as wearing the Fitbit on a given day if there was available heart rate data and greater than 1,500 steps taken, based on the criteria used in prior studies (33, 34). Average daily step count, active minutes per day, and very active minutes per day as determined by FitBit was calculated during baseline in both groups. For the intervention groups, these were calculated at the first, third, and sixth month as well; values per day were averaged for the course of the month. Compliance with exercise recommendations was discerned on the basis of the number of exercise classes attended, Fitbit use compliance, and Fitbit activity level analysis. Compliance with dietary recommendations were not tracked for this pilot study.

Nutrition data were acquired using the Block 2014 food frequency questionnaire (FFQ; NutritionQuest). This questionnaire includes a full-length FFQ for intake data, based on National Health and Nutrition Examination Survey (NHANES) dietary recall data. It has been validated across diverse populations (35, 36), and includes beverage and food data, with adjustments for fat, protein, carbohydrate, sugar, and whole grain. The FFQ was administered by an interviewer at baseline, 3 months, and 6 months. FFQ diet analysis output variables were then operationalized according to the combined ACS/American Institute of Cancer Research (AICR) dietary guidelines for cancer survivorship into seven scoring components (fruits/vegetables, whole grain/legumes, limit red meat, avoid processed meat, limit energy dense foods, avoid added sugars, limit alcohol; specific calculations detailed in Supplementary Table S1) rated on a scale from zero to three (none to highly adherent) and summed for a quantitative ACS/AICR total adherence index score, according to the methodology outlined by Springfield and colleagues (37). Glycemic index (GI) and glycemic load (GL) are FFQ diet analysis output variables. GI is obtained from United States Department of Agriculture (USDA) food groups derived from Food Patterns Equivalents Database (FPED) values for NHANES 2007–2010, and is reported as average daily GI. GL is calculated by multiplying the food-specific GI with the consumed quantity of that food daily, summed across food intake, and divided by 100.

### SOT 12-week Intervention

The intervention supported the adoption of the ACS nutrition and physical activity guidelines for cancer survivors: (i) consumption of a diet high in vegetables, fruits, and whole grains, and limited red meat and (ii) engaging in 150 minutes of moderate physical activity and two sessions of resistance exercise weekly (38). The intervention was grounded in SCT, which purports that behavior change is influenced by the dynamic interaction between personal (self-efficacy), behavioral (capability), and environmental (social support) factors. Self-monitoring and reinforcement are also critical (39). Studies, including ours, support SCT as a guiding framework for effective lifestyle interventions with cancer survivors (40–42). The intervention incorporated components from the evidence-based Moving Forward lifestyle program (32) as well as the Irwin and colleagues’ exercise trial (43). The SOT includes the following components:

- In-person one-on-one introductory meeting to review the intervention structure and provide materials (lifestyle pamphlet describing ACS guidelines, FitBit Charge HR with instructions).
- Three exercise sessions were conducted with a certified personal trainer (60 minutes, once per week, ideally during weeks 1–3) at a health club near the Radiation Oncology Department. The trainer guided participants in choosing appropriate moderate-intensity physical activities (based on the Borg Perceived Exertion Scale), supervised the participants as they engaged in 30 minutes of moderate activity, and then instructed them to perform a series of resistance exercises to build upper and lower lean tissue.
- Three dietary counseling sessions were conducted with licensed dieticians at the Froedtert and Medical College of Wisconsin (MCW) Cancer Center. During these sessions, they reviewed the ACS dietary guidelines and supported plant-based diets through small changes in daily consumption.
- Three weekly text messages (<200 characters) providing social support (e.g., “Look at what you've accomplished! Make a list of all you've changed and what keeps you going. Keep it somewhere to remind you how awesome you are”) and reinforcing adherence to exercise and diet guideline concordant behaviors (e.g., “Plant-based eating = focusing on foods that do not come from animals—vegetables, beans, fruits, whole grains. Each day think about how to eat the colors of the rainbow.”) were sent using the Mytapp platform, similar to our previous study (44). No personal health information was included in the texts.

In combination, the lifestyle pamphlet, personalized dietary and exercise counseling, and text messaging provided opportunities to improve knowledge, skills, and behavioral strategies to increase self-efficacy and behavioral capability to make lifestyle changes. The design also balanced time flexibility for participants with enough knowledge/guided sessions to encourage behavioral changes.

### Statistical Analysis

An intention-to-treat analysis was used to analyze the following outcomes: satisfaction questionnaire, Godin Leisure Physical Activity (PA) survey, Fitbit activity, Piper Fatigue Scale, Functional Assessment of Cancer Therapy-B (FACT-B) questionnaire, dual x ray absorptiometry (DEXA) body composition, circulating biomarkers, and FFQ (Supplementary Table S1). The outcomes were analyzed using a linear mixed-effects model. The fixed effects of group (intervention vs. control), time in months (0, 3, 6), and interaction of effects were included. Adjusted differences in the mean and estimated standard errors (SEs) are reported. Descriptive statistics were calculated for the patient demographics. Paired *t* tests, Wilcoxon rank-sum tests, and *χ*^2^ tests were used. The analyses were performed using SAS V9.4 (SAS Institute). A significance value of 0.05 was used. Significance values with Bonferroni adjustment for multiple comparisons are also reported in our Tables 2–6 as *P. *P* values noted in the Results are unadjusted *P* values; both adjusted and unadjusted *P* values are listed in Tables 2–6. The Bonferroni adjustment takes into account the multiple comparisons within model and not the fact there are many outcomes investigated, due to the exploratory nature of this study. This pilot study was not powered to find significant changes in outcome measures, but instead to assess (i) the feasibility of the intervention and (ii) trends in its impact on well-being, body composition, and biomarkers to guide broader implementation and study of SOT. Given our primary goal of identifying general trends for future hypothesis generation and the large number of simultaneous hypotheses tested, the adjusted *P* value might be overly conservative (45). Statistical analyses were performed with GraphPad Prism 9.1.2.

### Data Availability

The data generated in this study are available upon request from the corresponding author.

## Results

### Patient Characteristics

Of the 148 patients prescreened for eligibility, 45 met the eligibility criteria and consented to participate over a 16-month period. The primary reasons for ineligibility included treatment with mastectomy and comorbidities that prevented participation in the intervention components. The reasons for not opting for the study included time commitment, travel plans, and/or work conflicts. One patient withdrew prior to baseline evaluation, leaving 22 participants allocated to the intervention group and 22 to the control group. Participants were 57.0 ± 9.50 years of age and had a BMI of 31.8 ± 6.90; 1 of 22 (4.6%) patients in the control group had a BMI <25 (21.7) and 3 of 22 (13.6%) patients in the intervention group had a BMI <25 (20.9, 23.3, 22.5). The participants were predominantly white (79.5%), diagnosed with invasive disease (93.2%), and had not received chemotherapy (68.2%; Table 1). Most participants did not have CVD (95.5%) or CVD risk factors such as kidney disease (97.7%), high cholesterol (68.2%), or diabetes (84.1%). However, 47.7% of the participants had high blood pressure. There were no baseline differences in demographics, pre-existing medical conditions, tumor type/treatments, or education level between the control and intervention groups (Table 1). One patient was taken off study prior to completion due to a health issue deemed not related to the study. No adverse events related to the interventions were observed.

**TABLE 1:** Patient demographics at baseline

	Study ARM			
Variables	Total (*N* = 44) *N* (%)	Control (*N* = 22) *N* (%)	Intervention (*N* = 22) *N* (%)	*P* Value
Received chemo	14 (31.8)	8 (36.4)	6 (27.3)	*0.517*
Black or African American	3 (6.8)	0 (0.0)	3 (13.6)	*0.073*
Asian	2 (4.5)	1 (4.5)	1 (4.5)	*1.000*
White	35 (79.5)	19 (86.4)	16 (72.7)	*0.262*
American Indian or Alaska	0	0	0	*NA*
Native Hawaiian or Pacific Islander	0	0	0	*NA*
Hispanic or Latino	1 (2.3)	0 (0.0)	1 (4.5)	*0.312*
Non-Hispanic or Latino	34 (77.3)	18 (81.8)	16 (72.7)	*0.472*
Adults in household (Mean ± SD)	2.6 ± 0.92	2.2 ± 0.45	3 ± 1.1	*0.243*
What is the highest grade or year of school you completed?				*1.000*
Associate's degree or 2 year certificate	8 (72.7)	6 (75.0)	2 (66.7)	
High school graduate or GED	3 (27.3)	2 (25.0)	1 (33.3)	
Missing	33	14	19	
Age at the time of the study visit (Mean ± SD)	57.06 ± 9.52	58.15 ± 9.09	55.97 ± 10.03	*0.455*
BMI (Mean ± SD)	31.76 ± 6.85	32.27 ± 7.54	31.26 ± 6.26	*0.628*
Cancer type				*0.550*
Ductal carcinoma *In Situ* (DCIS)	3 (6.8)	2 (9.1)	1 (4.5)	
Invasive cancer	41 (93.2)	20 (90.9)	21 (95.5)	
Invasive cancer grade				*0.771*
1	21 (51.2)	11 (55.0)	10 (47.6)	
2	10 (24.4)	4 (20.0)	6 (28.6)	
3	10 (24.4)	5 (25.0)	5 (23.8)	
Estrogen receptor (+)	42 (95.5)	21 (95.5)	21 (95.5)	*1.000*
Progesterone receptor (+)	35 (79.5)	18 (81.8)	17 (77.3)	*0.709*
HER2 receptor (+)	4 (9.1)	2 (9.1)	2 (9.1)	*1.000*
Diabetes	7 (15.9)	4 (18.2)	3 (13.6)	*0.680*
High blood pressure	21 (47.7)	9 (40.9)	12 (54.5)	*0.365*
High cholesterol	14 (31.8)	7 (31.8)	7 (31.8)	*1.000*
Stroke/paralysis	1 (2.3)	1 (4.5)	0 (0.0)	*0.312*
Heart attack	1 (2.3)	0	1 (4.5)	*0.312*
Heart disease	2 (4.5)	0 (0.0)	2 (9.1)	*0.148*
Kidney disease	1 (2.3)	1 (4.5)	0 (0.0)	*0.312*
Emphysema/COPD	0	0	0	*NA*
Asthma	11 (25.0)	6 (27.3)	5 (22.7)	*0.728*
Arthritis/back problems	14 (31.8)	9 (40.9)	5 (22.7)	*0.195*
Tuberculosis	0	0	0	*NA*
Thyroid disorders	8 (18.2)	3 (13.6)	5 (22.7)	*0.434*
Blood disorders	2 (4.5)	1 (4.5)	1 (4.5)	*1.000*
Sleep apnea	8 (18.2)	5 (22.7)	3 (13.6)	*0.434*
Cancer	39 (88.6)	20 (90.9)	19 (86.4)	*0.635*

Abbreviation: COPD, chronic obstructive pulmonary disease.

### Feasibility, Participation, and Satisfaction

At baseline, 22 of 22 (100%) patients in the control group and 21 of 22 (95.4%) patients in the intervention group participated in body measurements, DEXA, labs, and questionnaires. At 3 months, 20 of 22 (90.9%) patients in the intervention group and 20 of 22 (90.9%) patients in the control group participated in body measurements, DEXA, labs, and questionnaires. At 6 months, 16 of 22 (72.7%) patients in the intervention group participated in the body measurements, DEXA, and labs, and 18 of 22 (81.8%) patients participated in the questionnaires. Within the control group, 20 of 22 (90.9%) participated in the body measurements, DEXA, and labs, and 17 of 22 (77.3%) participated in the questionnaires. Nineteen of the 22 (86.4%) participants in the intervention group participated in trainer-led exercise sessions. Eighteen of 22 (81.8%) participants completed at least one training session and 14 of 22 (63.6%) participants completed all three training sessions. During the baseline period, 18 of 22 (81.8%) participants in the intervention group and 10 of 22 (45.5%) participants in the control group wore the Fitbit for at least 6 days. Thirteen of 22 (59.1%) participants in the intervention group wore the Fitbit for >70% of the days of the SOT intervention. Of these participants, 11 of 13 (84.6%) continued to wear the Fitbit for at least 70% of the next 50 days, and 10 of 13 (76.9%) continued to wear the Fitbit for at least 70% of the full 6-month tracking period. Twelve of 13 (92.3%) of the participants who wore the Fitbit for >70% of the days during SOT attended at least one training session. In the control group, 5 of 22 (22.7%) participants continued to wear the Fitbit for >70% of the days during the SOT, even though they were not randomized to the Fitbit intervention. Of those 5 participants, 4 (80.0%) continued to wear the Fitbit for at least 70% of the next 50 days, and 4 (80.0%) continued to wear the Fitbit for at least 70% of the full 6-month tracking period.

Supplementary Table S3 details participant responses to the satisfaction survey administered during the 3-month follow-up. Fifteen (68.2%) participants in the intervention group and 15 (68.2%) in the control group completed the survey. Sixty-seven percent of participants in the intervention group (10/15) were “very satisfied” with the overall intervention, introductory program, personal training and dietary sessions, and Fitbit. Ninety-three percent (14/15) of the intervention group reported that Fitbit slightly or greatly helped them change their activity habits during the intervention, and 93% (14/15) reported that dietary counseling slightly or greatly helped them improve their dietary habits. Interestingly, only 33% of participants (5/15) were very satisfied with the text messages, and 40% (6/15) reported that text messages did not help them improve their activity habits. In addition, 40% of participants (6/15) reported that text messages did not help them improve their dietary habits.

In the control group, 47% (7/15) of the survey respondents were very satisfied with their study experience. Ninety-three percent (14/15) of them reported that participating in the trial slightly or greatly helped improve their activity levels, and 74% (11/15) felt that the trial slightly or greatly helped improve their dietary habits.

### Physical Activity and QoL

**FIGURE 2:**
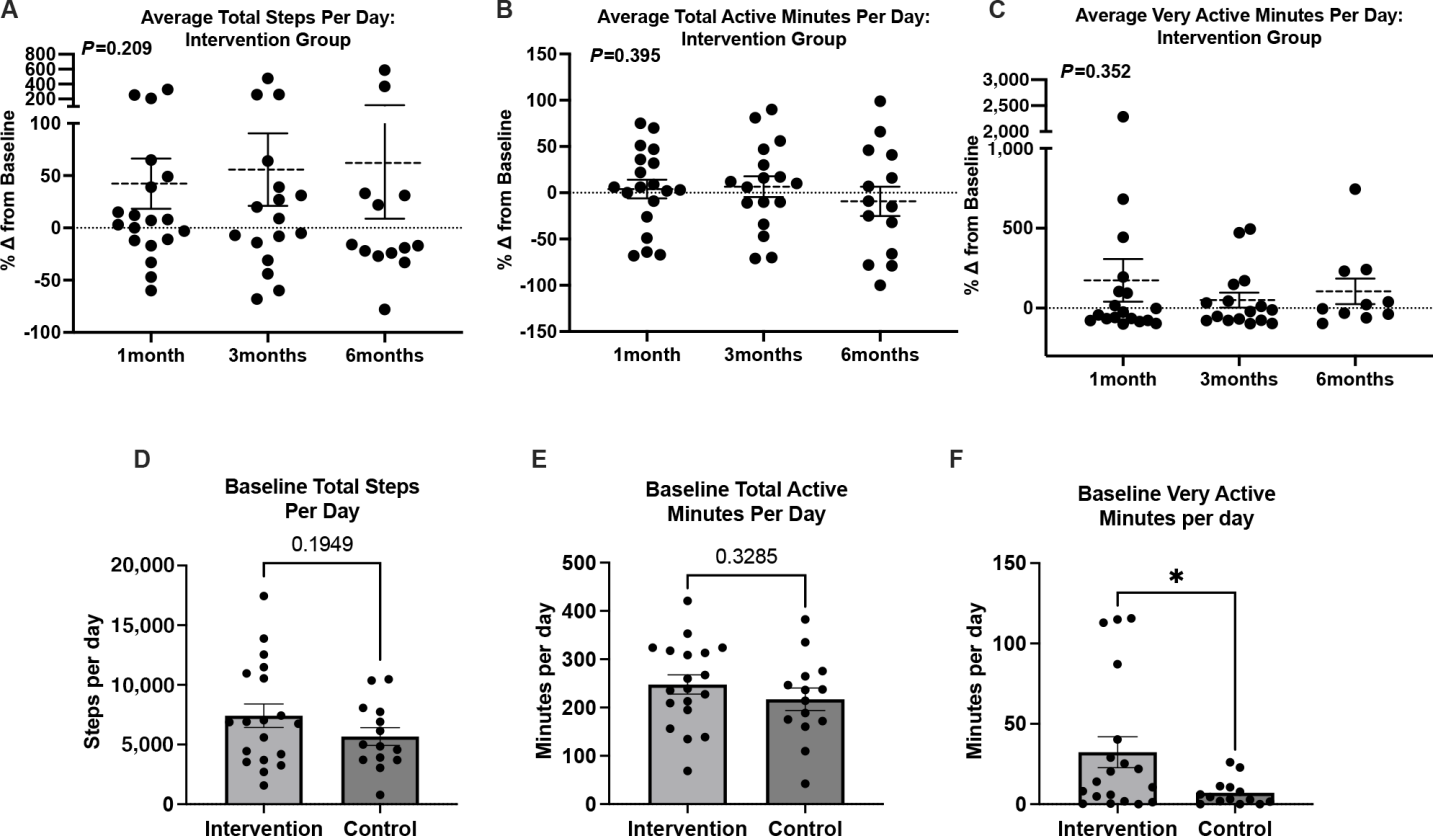
FitBit activity levels. Percent change in average total steps per day (**A**), average total active minutes per day (**B**), and average active minutes per day (**C**) in the intervention group compared with their baselines. Total steps per day (**D**), total active minutes per day (**E**), and total very active minutes per day (**F**) during the baseline week in the intervention and control groups. Graphs show mean ± SE. *, *P* < 0.05.

QoL was assessed using patient-reported outcomes in the Piper Fatigue Scale and FACT-B questionnaire. The Godin Leisure PA questionnaire was used to assess patient-reported physical activity levels. The results were compared within and between groups at baseline, 3 months, and 6 months (Table 2). Significant differences in some values were noted within groups over time. The control group showed a decrease in regular activity during leisure time (i.e., activity that results in sweating and rapid heart rates) at 6 months compared with baseline (−0.37 ± 0.16, *P* = 0.03), and the intervention group showed a decrease at 3 months (−0.35 ± 0.16, *P* = 0.04), but this was not sustained at 6 months (−0.21 ± 0.17, *P* = 0.22). The intervention group, but not the control group, showed an increase in total leisure activity at 3 months (21.97 ± 10.53, *P* = 0.04) compared with baseline, suggesting that even though they had a transient decrease in high-intensity activity, the intervention group overall increased the amount of time they stayed active. In line with these findings, evaluation of average daily total step count (Fig. 2A; % change from baseline to 1 month 42.3 ± 24.0, 3 months 55.8 ± 34.7, 6 months 62.1 ± 53.4; *P* = 0.21), active minutes (Fig. 2B; % change from baseline to 1 month 4.0 ± 10.1, 3 months 6.6 ± 11.3, 6 months −9.2 ± 15.9; *P* = 0.40), and very active minutes (Fig. 2C; % change from baseline to 1 month 173.7 ± 133.5, 3 months 49.8 ± 46.9, 6 months 104.7 ± 79.9 *P* = 0.35) recorded by the Fitbits in the intervention group showed that activity levels greatly varied across participants, but on average increased from their baseline and were maintained throughout the study. During the baseline period, no major differences in total step count (Fig. 2D; control 5,665 ± 744.3 steps vs. intervention 7,412 ± 987.7 steps, *P* = 0.20) and total active minutes (Fig. 2E; control 217.2 ± 23.3 minutes vs. intervention 247.7 ± 20.0 minutes, *P* = 0.33) were seen between intervention and control groups; however, the intervention group had greater very active minutes per day (Fig. 2F; control 7.1 ± 2.2 minutes vs. intervention 32.4 ± 9.7 minutes, *P* = 0.03).

**TABLE 2:** QoL measures and patient-reported physical activity at baseline, 3 months, and 6 months

	Intervention			Control			Intervention compared with Control		
		Estimate (SE) *P*-value			Estimate (SE) *P*-value			Estimate (SE) *P*-value	
Variable	Baseline Mean (SE)	Δ 3 months	Δ 6 months	Baseline Mean (SE)	Δ 3 months	Δ 6 months	Baseline Mean (SE)	Δ 3 months	Δ 6 months
Behavioral fatigue	2.32 (0.37)	0.12 (0.41) *P* = 0.778 *P = 1.000	0.11 (0.42) *P* = 0.804 *P = 1.000	3.37 (0.58)	0.34 (0.40) *P* = 0.396 *P = 1.000	0.08 (0.41) *P* = 0.837 *P = 1.000	−0.84 (0.65) *P* = 0.203	−1.06 (0.66) *P* = 0.112	−1.03 (0.66) *P* = 0.127
Affective meaning fatigue	3.55 (0.48)	0.07 (0.46) *P* = 0.883 *P = 1.000	−0.43 (0.47) *P* = 0.367 *P = 1.000	4.33 (0.60)	0.16 (0.45) *P* = 0.729 *P = 1.000	−0.26 (0.46) *P* = 0.576 *P = 1.000	−0.53 (0.76) *P* = 0.491	−0.62 (0.77) *P* = 0.428	−0.70 (0.78) *P* = 0.371
Sensory fatigue	4.02 (0.51)	0.10 (0.44) *P* = 0.818 *P = 1.000	−0.35 (0.45) *P* = 0.437 *P = 1.000	4.98 (0.56)	0.03 (0.43) *P* = 0.939 *P = 1.000	−0.46 (0.44) *P* = 0.296 *P = 0.889	−0.65 (0.76) *P* = 0.393	−0.58 (0.77) *P* = 0.451	−0.55 (0.77) *P* = 0.483
Cognitive/mood fatigue	3.48 (0.42)	−0.09 (0.31) *P* = 0.786 *P = 1.000	−0.26 (0.32) *P* = 0.430 *P = 1.000	3.80 (0.49)	0.24 (0.31) *P* = 0.441 *P = 1.000	0.18 (0.31) *P* = 0.559 *P = 1.000	−0.08 (0.63) *P* = 0.902	−0.40 (0.63) *P* = 0.529	−0.52 (0.64) *P* = 0.421
Overall fatigue (FACT-B)	3.34 (0.40)	0.05 (0.36) *P* = 0.887 *P = 1.000	−0.29 (0.37) *P* = 0.438 *P = 1.000	4.12 (0.51)	0.20 (0.35) *P* = 0.567 *P = 1.000	−0.10 (0.36) *P* = 0.779 *P = 1.000	−0.52 (0.65) *P* = 0.434	−0.67 (0.66) *P* = 0.317	−0.70 (0.67) *P* = 0.294
Regular activity at leisure time (with sweating and rapid heartbeat)	2.19 (0.16)	−0.35 (0.16) ***P* = 0.036** *P = 0.107	−0.21 (0.17) *P* = 0.216 *P = 0.649	2.29 (0.16)	−0.12 (0.16) *P* = 0.458 *P = 1.000	−0.37 (0.16) ***P* = 0.025** *P = 0.074	−0.10 (0.21) *P* = 0.643	−0.33 (0.22) *P* = 0.139	0.06 (0.22) *P* = 0.773
Total leisure activity	32.38 (4.97)	21.97 (10.53) ***P* = 0.041** *P = 0.122	11.34 (10.91) *P* = 0.302 *P = 0.907	34.64 (6.88)	9.06 (10.28) *P* = 0.381 *P = 1.000	13.98 (10.33) *P* = 0.180 *P = 0.541	−2.16 (11.54) *P* = 0.852	10.75 (12.07) *P* = 0.376	−4.80 (12.40) *P* = 0.699
Physical well being	3.32 (0.15)	−0.04 (0.14) *P* = 0.778 *P = 1.000	0.08 (0.14) *P* = 0.568 *P = 1.000	3.04 (0.18)	0.12 (0.14) *P* = 0.414 *P = 1.000	−0.06 (0.14) *P* = 0.677 *P = 1.000	0.15 (0.21) *P* = 0.479	−0.00 (0.22) *P* = 0.984	0.29 (0.22) *P* = 0.188
Social/family well being	3.07 (0.18)	.06 (0.11) *P* = 0.567 *P = 1.000	0.15 (0.11) *P* = 0.184 *P = 0.552	3.34 (0.14)	−0.36 (0.11) *P* = 0.002 *P = 0.005	−0.35 (0.11) *P* = 0.002 *P = 0.006	−0.31 (0.25) *P* = 0.224	0.12 (0.26) *P* = 0.653	0.19 (0.25) *P* = 0.456
Emotional well being	3.19 (0.13)	−0.11 (0.10) *P* = 0.270 *P = 0.810	−0.02 (0.10) *P* = 0.843 *P = 1.000	3.25 (0.13)	−0.05 (0.09) *P* = 0.617 *P = 1.000	−0.12 (0.09) *P* = 0.198 *P = 0.595	−0.08 (0.19) *P* = 0.667	−0.14 (0.20) *P* = 0.471	0.02 (0.20) *P* = 0.922
Functional well being	2.88 (0.18)	−0.03 (0.13) *P* = 0.792 *P = 1.000	0.10 (0.13) *P* = 0.460 *P = 1.000	2.66 (0.17)	0.11 (0.13) *P* = 0.372 *P = 1.000	0.22 (0.13) *P* = 0.085 *P = 0.255	0.18 (0.26) *P* = 0.485	0.03 (0.27) *P* = 0.898	0.06 (0.27) *P* = 0.827
Overall well being	3.09 (0.13)	−0.03 (0.09) *P* = 0.740 *P = 1.000	0.08 (0.09) *P* = 0.381 *P = 1.000	3.09 (0.12)	−0.05 (0.08) *P* = 0.584 *P = 1.000	−0.08 (0.08) *P* = 0.354 *P = 1.000	−0.01 (0.19) 0.9459	0.00 (0.19) *P* = 0.981	0.14 (0.19) *P* = 0.462
Overall pain score	5.48 (0.60)	0.17 (0.34) *P* = 0.615 *P = 1.000	0.03 (0.35) *P* = 0.920 *P = 1.000	6.05 (0.49)	−0.49 (0.33) *P* = 0.142 *P = 0.427	−0.14 (0.34) *P* = 0.674 *P = 1.000	−0.46 (0.80) *P* = 0.564	0.20 (0.81) *P* = 0.807	−0.29 (0.81) *P* = 0.726

**NOTE:** *P* = unadjusted *P* value; *P = adjusted *P* value with Bonferroni adjustment for multiple comparisons. Bold values are *P* < 0.05. Averages at 3 and 6 months have baseline mean subtracted for Δ 3 and Δ 6 months values. Intervention compared to Control was calculated using the difference between means of the two groups at each timepoint, with no need for multiple comparison adjustment due to two levels (Intervention/Control) with only one pairwise comparison.

From the FACT-B questionnaire, there was a significant decrease in social and family well-being at 3 months (−0.36 ± 0.11, *P* < 0.01) and 6 months (−0.35 ± 0.11, *P* < 0.01) compared with baseline in the control group, with no changes in the intervention group. There were no statistically significant differences within groups over time regarding fatigue, emotional/functional well-being, physical activity, or pain. There were no significant group x time interactions in any of the parameters in Table 2. In both groups, some of the commonly perceived reasons for fatigue included stress, poor sleep/lack of sufficient sleep, and treatment-related effects. Most participants (>75%) in both intervention and control groups reported that sleep, rest, and/or exercise helped most with relieving fatigue.

### Body Composition

DEXA scans for body composition, waist and hip circumference, and BMI were measured at baseline, 3 months, and 6 months (Table 3). Significant differences over time were observed for some measures within the groups. There was a nonstatistically significant trend toward reduced BMI in the intervention group, but not in the control group, at 6 months compared with baseline (−0.95 ± 0.49 cm, *P* = 0.06). In the intervention group, the average waist circumference also decreased at 3 months (−2.24 ± 1.01 cm, *P* = 0.03) and 6 months (−2.40 ± 1.10 cm, *P* = 0.03) compared with baseline, and hip circumference had a nonsignificant decreasing trend at 6 months (−1.68 ± 1.0 cm, *P* = 0.10). In the control group, waist circumference (−2.48 ± 1.09 cm, *P* = 0.03) and hip circumference (−2.39 ± 1.00 cm, *P* = 0.02) decreased at 6 months compared with baseline.

**TABLE 3:** Body composition and DEXA scan analysis at baseline, 3 months, and 6 months

	Intervention			Control			Intervention compared with Control		
		Estimate (SE) *P*-value			Estimate (SE) *P*-value			Estimate (SE) *P*-value	
Variable	Baseline Mean (SE)	Δ 3 months	Δ 6 months	Baseline Mean (SE)	Δ 3 months	Δ 6 months	Baseline Mean (SE)	Δ 3 months	Δ 6 months
BMI (kg/m^2^)	31.25 (1.48)	−0.71 (0.45) *P* = 0.121 *P = 0.363	−0.95 (0.49) *P* = 0.058 *P = 0.173	32.27 (1.51)	−0.46 (0.45) *P* = 0.311 *P = 0.933	−0.10 (0.49) *P* = 0.842 *P = 1.000	−0.74 (2.13) *P* = 0.729	−0.99 (2.14) *P* = 0.644	−1.59 (2.15) *P* = 0.462
Ideal body weight (kg)	53.61 (0.91)	0.05 (1.39) *P* = 0.972 *P = 1.000	−0.05 (1.51) *P* = 0.972 *P = 1.000	55.58 (1.42)	−2.17 (1.38) *P* = 0.120 *P = 0.360	−1.15 (1.49) *P* = 0.441 *P = 1.000	−1.89 (2.36) *P* = 0.426	0.33 (2.41) *P* = 0.891	−0.79 (2.52) *P* = 0.755
Avg waist circumference (cm)	101.10 (3.46)	−2.24 (1.01) ***P* = 0.030** *P = 0.089	−2.40 (1.10) ***P* = 0.032** *P = 0.096	104.53 (4.10)	−0.92 (1.02) *P* = 0.369 *P = 1.000	−2.48 (1.09) ***P* = 0.026** *P = 0.079	−2.70 (5.39) *P* = 0.619	−4.01 (5.41) *P* = 0.461	−2.62 (5.43) *P* = 0.632
Avg hip circumference (cm)	111.96 (2.78)	−1.24 (0.92) *P* = 0.183 *P = 0.549	−1.68 (1.00) *P* = 0.098 *P = 0.294	114.41 (3.23)	−1.34 (0.93) *P* = 0.154 *P = 0.462	−2.39 (1.00) ***P* = 0.019** *P = 0.058	−1.77 (4.27) *P* = 0.679	−1.67 (4.28) *P* = 0.698	−1.06 (4.31) *P* = 0.806
Total mass (kg)	80.89 (3.96)	−1.88 (0.80) ***P* = 0.022** *P = 0.067	−2.37 (0.87) *P* = 0.008 *P = 0.025	85.69 (4.57)	−1.32 (0.80) *P* = 0.103 *P = 0.308	−2.69 (0.87) *P* = 0.003 *P = 0.009	−4.05 (5.95) *P* = 0.499	−4.60 (5.96) *P* = 0.442	−3.73 (5.98) *P* = 0.534
Tissue mass (g)	78561.19 (3919.72)	−1865.63 (802.02) ***P* = 0.023** *P = 0.069	−2346.03 (873.82) *P* = 0.009 *P = 0.027	83259.95 (4676.91)	−1298.01 (797.26) *P* = 0.108 *P = 0.324	−2658.78 (869.40) *P* = 0.003 *P = 0.010	−3962.49 (5877.3) *P* = 0.503	−4530.10 (5884.1) *P* = 0.444	−3649.74 (5901.2) *P* = 0.538
Fat-free mass (g)	45038.05 (1370.77)	−895.72 (359.98) *P* = 0.015 *P = 0.046	−1059.01 (392.16) *P* = 0.009 *P = 0.026	46603.41 (1832.46)	−623.16 (357.83) *P* = 0.086 *P = 0.258	−721.37 (390.10) *P* = 0.069 *P = 0.206	−1227.62 (2144.7) *P* = 0.569	−1500.19 (2148.5) *P* = 0.487	−1565.27 (2157.9) *P* = 0.471
Fat mass (g)	35846.24 (2926.50)	−980.34 (615.78) *P* = 0.116 *P = 0.348	−1316.73 (670.88) *P* = 0.054 *P = 0.161	39075.59 (2988.97)	−691.00 (612.12) *P* = 0.263 *P = 0.789	−1965.99 (667.44) *P* = 0.004 *P = 0.013	−2812.76 (4149.9) *P* = 0.500	−3102.09 (4155.5) *P* = 0.458	−2163.49 (4169.8) *P* = 0.606
Lean mass (g)	42715.05 (1326.41)	−882.46 (359.74) ***P* = 0.017** *P = 0.050	−1042.61 (391.89) *P* = 0.009 *P = 0.029	44184.27 (1686.97)	−611.37 (357.59) *P* = 0.092 *P = 0.276	−696.89 (389.81) *P* = 0.078 *P = 0.235	−1154.36 (2059.2) 0.577	−1425.45 (2063.1) 0.492	−1500.07 (2072.9) 0.472
Estimated visceral fat (%)	31.47 (3.24)	−2.17 (1.22) *P* = 0.081 *P = 0.243	−1.73 (1.33) *P* = 0.198 *P = 0.595	32.73 (2.39)	0.73 (1.27) *P* = 0.568 *P = 1.000	−1.68 (1.36) *P* = 0.222 *P = 0.665	−1.41 (4.18) *P* = 0.738	−4.31 (4.21) *P* = 0.311	−1.45 (4.26) *P* = 0.734

NOTE: *P* = unadjusted *P* value; *P = adjusted *P* value with Bonferroni adjustment for multiple comparisons. Bold values are *P* < 0.05. Averages at 3 and 6 months have baseline mean subtracted for Δ 3 and Δ 6 months values. Intervention compared with Control was calculated using the difference between means of the two groups at each timepoint, with no need for multiple comparison adjustment due to two levels (Intervention/Control) with only one pairwise comparison.

Total body mass decreased in the intervention group at 3 months (−1.88 ± 0.80 kg, *P* = 0.02) and 6 months (−2.37 ± 0.87 kg, *P* < 0.01), and in the control group at 6 months (−2.69 ± 0.87 kg, *P* < 0.01) compared with baseline. The total tissue mass decreased at 6 months (intervention: −2346.0 ± 837.8 g, *P* = 0.01; control: −2658.8 ± 869.4 g, *P* < 0.01) compared with baseline in both groups and at 3 months in the intervention group (−1865.6 ± 802.02 g, *P* = 0.02).

The total fat mass was significantly lower at 6 months (−1965.99 ± 667.44 g, *P* < 0.01) than at baseline in the control group only, with a nonstatistically significant trend toward decreased fat mass in the intervention group (−1316.73 ± 670.88 g, *P* = 0.05). The total fat-free mass decreased at 3 months (−895.72 ± 359.98 g, *P* = 0.02) and 6 months (−1059.01 ± 392.16 g, *P* = 0.01) in the intervention group. In addition, only the intervention group showed a decrease in lean mass at 3 months (−882.46 ± 357.74 g, *P* = 0.02) and 6 months (−1042.61 ± 391.87 g, *P* = 0.01), although a nonstatistically significant trend toward reduction was also noticed in the control group (6 months; −696.89 ± 389.81 g, *P* = 0.08). The intervention group also showed a nonstatistically significant trend toward a reduction in estimated visceral fat at 3 months (−2.17 ± 1.22%, *P* = 0.08) compared with baseline, with no differences in the control group. There were no significant group x time interactions in any of these measures.

### RMR and Biomarkers

RMR was calculated using the Harris–Benedict (46) and Owen equations (47), with no differences at baseline between the groups. There was a decrease in RMR at 3 months (Harris −19.54 ± 7.94, *P* = 0.02; Owen −13.17 ± 5.63, *P* = 0.02) and 6 months (Harris −25.03 ± 8.65, *P* = 0.01; Owen −16.68 ± 6.14, *P* = 0.01) compared with baseline in the intervention group, and a decrease at 6 months (Harris −27.71 ± 8.61, *P* < 0.01; Owen −17.15 ± 6.11, *P* = 0.01) compared with baseline in the control group (Table 4). The control group had an estimated increase in HbA1C at 3 months compared with baseline (0.26 ± 0.10%, *P* = 0.01), with a nonstatistically significant trend toward increase at 6 months (0.22 ± 0.13%, *P* = 0.09). The control group also showed an increase in glucose levels at 6 months (8.42 ± 3.94 mg/dL, *P* = 0.04). In the intervention group, there were no differences in HbA1C, glucose, or insulin levels between baseline, 3 months, and 6 months. Leptin levels decreased significantly at 6 months (−13583.7 ± 6184.2 pg/mL, *P* = 0.03) compared with baseline in the control group only. Adiponectin levels decreased at 3 months (control: −3072.35 ± 1117.9 pg/mL, *P* = 0.01; intervention: −2671.20 ± 1129.67 pg/mL, *P* = 0.02) and 6 months (control: −5909.32 ± 1136.3 pg/mL, *P* < 0.01; intervention –5252.58 ± 1176.84 pg/mL, *P* < 0.01) compared with baseline in both groups. There were no significant differences in leptin:adiponectin ratios between the groups or at different timepoints within the groups. There were no significant group x time interactions across these measures.

**TABLE 4:** Comparisons of resting metabolism and energy expenditure, and metabolic biomarkers at baseline, 3 months, and 6 months

	Intervention			Control			Intervention compared with Control		
		Estimate (SE) *P*-value			Estimate (SE) *P*-value			Estimate (SE) *P*-value	
Variable	Baseline Mean (SE)	Δ 3 months	Δ 6 months	Baseline Mean (SE)	Δ 3 months	Δ 6 months	Baseline Mean (SE)	Δ 3 months	Δ 6 months
Resting metabolic rate comparisons									
Predicted Harris comparison	1482.76 (42.87)	−19.54 (7.94) *P* = 0.016 *P = 0.050	−25.03 (8.65) ***P* = 0.005** *P = 0.150	1521.96 (47.15)	−11.71 (7.89) *P* = 0.143 *P = 0.428	−27.71 (8.61) *P* = 0.002 *P = 0.006	−28.27 (63.68) *P* = 0.659	−36.11 (63.75) *P* = 0.573	−25.59 (63.90) *P* = 0.690
Predicted Owen comparison	1379.52 (28.84)	−13.17 (5.63) ***P* = 0.022** *P = 0.067	−16.86 (6.14) *P* = 0.008 *P = 0.023	1415.68 (33.41)	−8.56 (5.60) *P* = 0.131 *P = 0.393	−17.15 (6.11) *P* = 0.007 *P = 0.020	−30.33 (43.88) *P* = 0.492	−34.93 (43.92) *P* = 0.429	−30.03 (44.04) *P* = 0.498
Metabolic biomarkers									
HgA1C (%)	5.83 (0.20)	0.03 (0.10) *P* = 0.784 *P = 1.000	0.03 (0.1) *P* = 0.812 *P = 1.000	5.54 (0.14)	0.26 (0.10) ***P* = 0.012** *P = 0.037	0.22 (0.13) *P* = 0.086 *P = 0.257	0.31 (0.23) *P* = 0.189	0.07 (0.24) *P* = 0.759	0.12 (0.26) *P* = 0.658
Glucose (mg/dL)	100 (6.64)	−0.83 (3.66) *P* = 0.822 *P = 1.000	−2.70 (3.76) *P* = 0.476 *P = 1.000	100.76 (4.93)	5.22 (3.28) *P* = 0.117 *P = 0.351	8.42 (3.94) ***P* = 0.037** *P = 0.111	−0.76 (8.74) *P* = 0.931	−6.81 (8.97) *P* = 0.451	−11.88 (9.27) *P* = 0.206
Insulin (pmol/L)	77.69 (11.55)	−1.46 (9.81) *P* = 0.881 *P = 1.000	−12.81 (10.21) *P* = 0.214 *P = 0.642	90.24 (11.34)	−10.83 (9.71) *P* = 0.269 *P = 0.806	−14.30 (9.85) *P* = 0.151 *P = 0.454	−11.28 (16.34) *P* = 0.492	−1.92 (16.87) *P* = 0.910	−9.80 (17.09) *P* = 0.568
Leptin (pg/mL)	51893.81 (8043.5)	−3395.81 (6151.9) *P* = 0.583 *P = 1.000	−828.55 (6406.8) *P* = 0.898 *P = 1.000	60656 (7600.8)	−6626.05 (6089.4) *P* = 0.280 *P = 0.841	−13583.7 (6184.2) ***P* = 0.031** *P = 0.094	−7210.18 (10977) *P* = 0.514	−3979.93 (11294) *P* = 0.726	5544.97 (11427) *P* = 0.629
Adiponectin (pg/mL)	17887.62 (1463.2)	−2671.20 (1129.67) ***P* = 0.021** *P = 0.063	−5252.58 (1176.84) p<0.0001 *p<0.0001	15223 (1753.1)	−3072.35 (1117.9) *P* = 0.008 *P = 0.023	−5909.32 (1136.3 p<0.0001 *p<0.0001	2486.67 (2187.7) *P* = 0.260	2887.82 (2242.5) *P* = 0.202	3143.42 (2265.1) *P* = 0.170
Leptin: Adiponectin ratio	3.70 (0.74)	3.65 (1.96) *P* = 0.068 *P = 0.203	2.48 (2.00) *P* = 0.220 *P = 0.659	5.75 (1.14)	1.06 (1.91) *P* = 0.581 *P = 1.000	2.56 (1.93) *P* = 0.189 *P = 0.567	−1.89 (2.61) *P* = 0.471	0.70 (2.75) *P* = 0.801	−1.97 (2.78) *P* = 0.480

**NOTE:** *P* = unadjusted *P* value; *P = adjusted *P* value with Bonferroni adjustment for multiple comparisons. Bold values are *P* < 0.05. Averages at 3 and 6 months have baseline mean subtracted for Δ 3 and Δ 6 months values. Intervention compared with Control was calculated using the difference between means of the two groups at each timepoint, with no need for multiple comparison adjustment due to two levels (Intervention/Control) with only one pairwise comparison.

### Inflammatory Markers

No baseline differences in cytokine and inflammatory marker levels were observed between the control and intervention groups. There was a decrease in the levels of the anti-inflammatory IL1 receptor antagonist (IL1RA) at 3 months compared with baseline in the control group (−4.92 ± 1.24 mg/L, *P* = 0.01) and a nonstatistically significant trend toward decrease in the intervention group (−2.44 ± 1.31 mg/L, *P* = 0.05; Table 5). At 6 months, only the controls continued to show a decrease in IL1RA levels (−3.46 ± 1.24 mg/L, *P* = 0.01). GMCSF (−98.27 ± 38.44 mg/L, *P* = 0.01) and IL1a (−2.44 ± 10.57 mg/L, *P* = 0.04) decreased at 3 months compared with baseline in the control group, with no differences in the intervention group. In the intervention group, IL8 levels decreased at 6 months (−2.77 ± 1.51 mg/L, *P* = 0.02) compared with baseline. There were no differences in IL1b, IL2, IL10, IL12p, or TNFα levels within or between groups (Table 5). Of note, there were no significant group x time interactions in the cytokine data.

**TABLE 5:** Comparisons of cytokine levels at baseline, 3 months, and 6 months

	Intervention			Control			Intervention compared with Control		
		Estimate (SE) *P*-value			Estimate (SE) *P*-value			Estimate (SE) *P*-value	
Variable	Baseline Mean (SE)	Δ 3 months	Δ 6 months	Baseline Mean (SE)	Δ 3 months	Δ 6 months	Baseline Mean (SE)	Δ 3 months	Δ 6 months
C-reactive protein (mg/L)	4.25 (1.11)	−0.26 (0.55) *P* = 0.636 *P = 1.000	−0.70 (0.59) *P* = 0.242 *P = 0.726	3.67 (0.63)	0.46 (0.52) *P* = 0.379 *P = 1.000	−0.37 (0.60) *P* = 0.541 *P = 1.000	0.59 (1.18) *P* = 0.622	−0.13 (1.21) *P* = 0.912	0.26 (1.27) *P* = 0.839
IL1RA (mg/L)	6.28 (1.01)	−2.44 (1.23) *P* = 0.051 *P = 0.152	−1.75 (1.31) *P* = 0.186 *P = 0.558	14.08 (5.57)	−4.92 (1.24) *P* = 0.002 *p = 0.005	−3.46 (1.24) *P* = 0.007 *P = 0.020	−7.41 (5.24) *P* = 0.163	−4.93 (5.28) *P* = 0.354	−5.69 (5.29) *P* = 0.286
GMCSF (mg/L)	43.46 (29.84)	3.09 (38.09) *P* = 0.936 *P = 1.000	−17.76 (40.68) *P* = 0.664 *P = 1.000	225.12 (195.39)	−98.27 (38.44) *P* = 0.013 *P = 0.039	−44.71 (38.44) *P* = 0.249 *P = 0.747	−174.05 (14739.4) *P* = 0.991	−72.69 (14739.4) *P* = 0.996	−147.10 (14739.4) *P* = 0.992
IFNγ (mg/L)	13.27 (4.60)	5.52 (60.22) *P* = 0.927 *P = 1.000	0.60 (64.23 *P* = 0.993 *P = 1.000	210.36 (190.56)	−116.78 (60.80) *P* = 0.059 *P = 0.177	−91.90 (60.65) *P* = 0.134 *P = 0.403	−192.72 (143.15) *P* = 0.183	−70.42 (146.12) *P* = 0.631	−100.21 (147.24) *P* = 0.490
IL1a (mg/L)	18.99 (7.17)	−7.44 (10.47) *P* = 0.480 *P = 1.000	−4.74 (11.17) *P* = 0.673 *P = 1.000	60.06 (30.93)	−22.73 (10.57) ***P* = 0.035** *P = 0.106	−7.37 (10.55) *P* = 0.487 *P = 1.000	−39.07 (29.47) *P* = 0.190	−23.78 (29.91) *P* = 0.430	−36.45 (30.08) *P* = 0.230
IL1b (mg/L)	12.58 (4.30)	−3.96 (5.95) *P* = 0.508 *P = 1.000	−1.44 (6.35) *P* = 0.821 *P = 1.000	32.33 (15.76)	−9.15 (6.01) *P* = 0.132 *P = 0.397	1.42 (6.00) *P* = 0.813 *P = 1.000	−18.43 (17.26) *P* = 0.290	−13.24 (17.51) *P* = 0.452	−21.30 (17.60) *P* = 0.231
IL2 (mg/L)	1.26 (0.71)	−0.50 (1.60) *P* = 0.757 *P = 1.000	−0.41 (1.70) *P* = 0.809 *P = 1.000	7.59 (5.37)	1.12 (1.61) *P* = 0.490 *P = 1.000	2.50 (1.61) *P* = 0.125 *P = 0.375	−5.90 (6.88) *P* = 0.394	−7.52 (6.93) *P* = 0.282	−8.82 (6.95) *P* = 0.209
IL6 (mg/L)	2.66 (0.60)	−0.76 (3.60) *P* = 0.833 *P = 1.000	−0.80 (3.84) *P* = 0.835 *P = 1.000	11.69 (9.17)	−6.19 (3.64) *P* = 0.094 *P = 0.281	−6.26 (3.62) *P* = 0.089 *P = 0.266	−8.87 (6.45) *P* = 0.174	−3.45 (6.67) *P* = 0.607	−3.42 (6.76) *P* = 0.615
IL8 (mg/L)	7.93 (2.16)	−1.62 (1.08) *P* = 0.138 *P = 0.414	−2.77 (1.15) ***P* = 0.019** *P = 0.057	7.63 (1.09)	−0.14 (1.09) *P* = 0.896 *P = 1.000	0.02 (1.09) *P* = 0.988 *P = 1.000	0.31 (1.88) *P* = 0.870	−1.17 (1.94) *P* = 0.549	−2.47 (1.97) *P* = 0.214
IL10 (mg/L)	2.75 (1.11)	−0.34 (1.86) *P* = 0.855 *P = 1.000	−0.12 (1.98) *P* = 0.953 *P = 1.000	9.17 (6.33)	−2.98 (1.88) *P* = 0.116 *P = 0.349	−0.03 (1.87) *P* = 0.988 *P = 1.000	−6.05 (5.58) *P* = 0.282	−3.41 (5.65) *P* = 0.548	−6.14 (5.68) *P* = 0.283
IL12 (mg/L)	4.38 (1.27)	1.01 (5.36) *P* = 0.852 *P = 1.000	4.28 (5.73) *P* = 0.457 *P = 1.00	35.04 (28.20)	−7.30 (5.41) *P* = 0.182 *P = 0.546	3.81 (5.41) *P* = 0.484 *P = 1.000	−29.03 (27.76) *P* = 0.299	−20.72 (27.89) *P* = 0.460	−28.55 (27.93) *P* = 0.310
TNFa (mg/L)	54.38 (12.30)	−0.39 (72.30) *P* = 0.9957 *P = 1.000	−0.65 (77.14) *P* = 0.9933 *P = 1.000	329.25 (245.27)	−143.40 (73.00) *P* = 0.0536 *P = 0.161	−91.49 (72.85) *P* = 0.2135 *P = 0.641	−267.15 (190.41) *P* = 0.165	−124.14 (193.67) *P* = 0.524	−176.32 (194.89) *P* = 0.369

NOTE: *P* = unadjusted *P* value; *P = adjusted *P* value with Bonferroni adjustment for multiple comparisons. Bold values are *P* < 0.05. Averages at 3 and 6 months have baseline mean subtracted for Δ 3 and Δ 6 months values. Intervention compared with Control was calculated using the difference between means of the two groups at each timepoint, with no need for multiple comparison adjustment due to two levels (Intervention/Control) with only one pairwise comparison.

### Nutrition

The FFQ was completed in the intervention group by 22 of 22 (100%) participants at baseline, 11 of 22 (50.0%) participants at 3 months, and 15 of 22 (68.2%) participants at 6 months. The FFQ was completed in the control group by 19 of 22 (86.4%) participants at baseline, 16 of 22 (72.7%) participants at 3 months, and 15 of 22 (68.2%) participants at 6 months. FFQ data are presented in Table 6. There was a statistically significant, though clinically modest, decrease in GI at 3 months compared with baseline in the intervention group (−2.0 ± 0.96, *P* = 0.03), not seen in the control group. In the control group at 6 months, there was a significant reduction in GL compared with baseline (−28.05 ± 7.47, *P* < 0.01), and the control group also had increased adherence to avoiding added sugars at 6 months (0.61 ± 0.25, *P* = 0.02). The intervention group had statistically significant improvements in adherence for “limit alcohol” at 6 months (0.18 ± 0.08, *P* = 0.03), an effect not seen in the control group. The control group had reduced adherence with “eat variety of fruits and vegetables” at 6 months (−0.55 ± 0.23, *P* = 0.02), with no significant changes over time in the intervention group. The intervention group had improved adherence with eating whole grains/legumes at 3 months (0.73 ± 0.26, *P* < 0.01), with no major changes over time in the control group. There was a statistically significant improvement in the ACS/AICR score at 3 months versus baseline (1.50 ± 0.56, *P* = 0.01) in the intervention group that was no longer seen at 6 months. There were no statistically significant changes in ACR/AICR score seen in the control group at 3 or 6 months compared with baseline.

**TABLE 6:** Comparisons of nutritional outcomes at baseline, 3 months, and 6 months

	Intervention			Control			Intervention compared with Control		
		Estimate (SE) *P*-value			Estimate (SE) *P*-value			Estimate (SE) *P*-value	
Variable	Baseline Mean (SE)	Δ 3 months	Δ 6 months	Baseline Mean (SE)	Δ 3 months	Δ 6 months	Baseline Mean (SE)	Δ 3 months	Δ 6 months
Glycemic index	50.22 (0.69)	−2.19 (0.96) ***P* = 0.028** *P = 0.083	−0.62 (0.86) *P* = 0.475 *P = 1.000	51.58 (1.01)	−1.35 (0.90) *P* = 0.137 *P = 0.411	−1.21 (0.90) *P* = 0.187 *P = 0.561	−1.44 (1.12) *P* = 0.206	−2.27 (1.31) *P* = 0.089	−0.84 (1.25) *P* = 0.504
Glycemic load	85.81 (1.01)	−1.47 (8.01) *P* = 0.855 *P = 1.000	−3.04 (7.11) *P* = 0.671 *P = 1.000	79.48 (1.16)	−7.36 (7.42) *P* = 0.326 *P = 0.977	−28.05 (7.47) *P* < 0.001 *P = 0.001	10.34 (10.67) *P* = 0.337	16.24 (12.07) *P* = 0.185	35.35 (11.66) ***P* = 0.004**
Fruits/Vegetables	2.09 (0.10)	0.45 (0.24) *P* = 0.073 *P = 0.220	0.13 (0.22) *P* = 0.539 *P = 1.000	1.95 (0.13)	−0.17 (0.22) *P* = 0.465 *P = 1.000	−0.55 (0.23) ***P* = 0.021** *P = 0.062	0.16 (0.26) *P* = 0.529	0.78 (0.31) ***P* = 0.015**	0.85 (0.29) ***P* = 0.006**
Whole Grains/Legumes	1.95 (0.22)	0.73 (0.26) *P* = 0.007 *P = 0.021	0.04 (0.23) *P* = 0.868 *P = 1.000	0.47 (0.05)	−0.03 (0.24) *P* = 0.908 *P = 1.000	0.09 (0.24) *P* = 0.724 *P = 1.00	0.65 (0.31) ***P* = 0.039**	1.41 (0.36) ***P* < 0.001**	0.65 (0.31) *P* = 0.083
Limit red meat	3.00 (0.00)	0.00 (0.07) *P* = 1.000 *P = 1.000	0.00 (0.07) *P* = 1.000 *P = 1.000	2.90 (0.04)	0.11 (0.07) *P* = 0.140 *P = 0.420	0.11 (0.07) *P* = 0.148 *P = 0.443	0.11 (0.06) *P* = 0.104	0.00 (0.08) *P* = 1.000	0.00 (0.07) *P* = 1.000
Avoid processed meat	2.81 (0.01)	−0.08 (0.13) *P* = 0.527 *P = 1.000	0.00 (0.11) *P* = 0.996 *P = 1.000	2.79 (0.02)	0.12 (0.12) *P* = 0.315 *P = 0.945	0.10 (0.12) *P* = 0.384 *P = 1.000	0.04 (0.16) *P* = 0.789	−0.16 (0.18) *P* = 0.393	−0.06 (0.18) *P* = 0.731
Limit energy dense foods	2.32 (0.04)	−0.17 (0.24) *P* = 0.481 *P = 1.000	−0.07 (0.22) *P* = 0.753 *P = 1.000	2.47 (0.16)	−0.27 (0.23) *P* = 0.250 *P = 0.749	−0.47 (0.24) *P* = 0.054 *P = 0.161	−0.16 (0.21) *P* = 0.443	−0.06 (0.26) *P* = 0.818	0.24 (0.24) *P* = 0.324
Avoid added sugars	1.86 (0.03)	0.08 (0.27) *P* = 0.770 *P = 1.000	0.09 (0.24) *P* = 0.704 *P = 1.000	1.89 (0.20)	0.20 (0.25) *P* = 0.433 *P = 1.000	0.61 (0.25) ***P* = 0.020** *P = 0.061	−0.15 (0.33) *P* = 0.646	−0.27 (0.38) *P* = 0.480	−0.67 (0.33) *P* = 0.076
Limit alcohol	1.82 (0.17)	0.18 (0.09) *P* = 0.051 *P = 0.171	0.18 (0.08) ***P* = 0.032** *P = 0.096	2.05 (0.06)	0.06 (0.09) *P* = 0.462 *P = 1.000	0.14 (0.09) *P* = 0.104 *P = 0.312	−0.19 (0.24) *P* = 0.448	−0.07 (0.25) *P* = 0.561	−0.15 (0.25) *P* = 0.795
ACS/AICR score	14.95 (0.75)	1.50 (0.56) *P* = 0.010 *P = 0.029	0.46 (0.50) *P* = 0.358 *P = 1.000	14.58 (1.04)	0.11 (0.52) *P* = 0.830 *P = 1.000	−0.01 (0.52) *P* = 0.991 *P = 1.000	0.49 (0.66) *P* = 0.460	1.88 (0.77) ***P* = 0.018**	0.96 (0.74) *P* = 0.199

NOTE: *P* = unadjusted *P* value; *P = adjusted *P* value with Bonferroni adjustment for multiple comparisons. Bold values are *P* < 0.05. Averages at 3 and 6 months have baseline mean subtracted for Δ 3 and Δ 6 months values. Intervention compared with Control was calculated using the difference between means of the two groups at each timepoint, with no need for multiple comparison adjustment due to two levels (Intervention/Control) with only one pairwise comparison.

When comparing within timepoints between groups, the ACS/AICR score was increased in the intervention group at 3 months compared with control (1.88 ± 0.77, *P* = 0.02). For component scores, there was better adherence in the intervention versus the control group for “eat variety of fruits and vegetables” at 3 months (0.78 ± 0.31, *P* = 0.02) and 6 months (0.85 ± 0.29, *P* < 0.01), and for “eat whole grains and legumes” at 3 months (1.41 ± 0.36, *P* < 0.01), although adherence to eating whole grains and legumes was higher within the intervention group compared with the control group at baseline (0.65 ± 0.31, *P* = 0.04). Interestingly, compared with the control group, the intervention group had an increase in GL at 6 months (35.35 ± 11.66, *P* < 0.01).

## Discussion

While radiotherapy is a crucial treatment for many patients with breast cancer, fatigue and other radiotherapy-related symptoms can lead to sedentary behavior, physical deconditioning, muscle wasting, and loss of function, which can contribute to worse QoL and increased CVD risk among patients with breast cancer (1, 2). Recent studies highlight that exercise is safe and effective during active treatment; it may further improve cardiovascular outcomes, combat side effects, and enhance QoL (48, 49). To our knowledge, our pilot is one of the first prospective combined diet and exercise lifestyle interventions during radiotherapy for women with breast cancer employing electronic personal fitness tracking. Furthermore, our study incorporates patient-reported outcomes, objective body mass data, blood markers, Fitbit activity data, and FFQs to better assess the intervention, and it is focused on women with an existing BMI of greater than or equal to 25 or those not adhering to the ACS dietary and activity guidelines; this group is associated with worse cancer-related outcomes compared with women within normal BMI ranges (50). The high level of participation in surveys and intervention sessions, the large percentage of Fitbit usage in the intervention group, and the positive feedback from participants in the intervention group demonstrated that the SOT program is both feasible and yields promising results.

Prior studies in patients with cancer have reported poor adherence to Fitbit usage, with improved engagement when reminders (texts/emails) are included (51–54). Our study incorporated both the Fitbit and text reminders. A total of 59% of the participants in the intervention group adhered to wearing the Fitbit for more than 70% of the days during the SOT intervention. Other studies employing Fitbit have reported adherence rates of 16%–91% (51–54); given the variability in patient populations, study design, and criteria for adherence measurements, we were unable to directly compare our outcomes. Nonetheless, results from the satisfaction survey demonstrated that 94% of respondents felt that the Fitbit helped them improve their activity levels during the study. Interestingly, while prior studies have suggested that text reminders are important for ensuring adherence to Fitbit use/activity recommendations, our respondents reported poor satisfaction with the text reminders and felt that they did not help improve their activity or dietary habits. However, the intervention group self-reported an increase in physical activity. This suggests that perhaps the text message reminders were inconvenient but still helpful in ensuring patient adherence to intervention.

The SOT intervention did not affect fatigue or emotional, functional, or physical well-being. However, the control group reported a significant decrease in social and family well-being, which was not observed among intervention participants. Our findings contrast with those of Carayol and colleagues, who noted improvements in fatigue following diet and exercise interventions in patients with breast cancer undergoing chemotherapy and radiotherapy (26). Whether this discrepancy is due to differences in exercise regimen (Carayol and colleagues’ study prescribed lower weekly exercise doses), lower BMI of study participants (mean of 25 kg/m^2^), differences in intervention design, receipt of both chemotherapy and radiotherapy in their study, and/or lack of statistical power in our pilot study is unknown. Other studies support robust improvements in QoL with higher levels of physical activity in overweight/obese patients with breast cancer (55). Moreover, behavioral factors such as sleep dysfunction and enjoyment of physical activity influence fatigue outcomes following interventions (56). While these were not assessed in the current study, the maintained QoL in our pilot study of the SOT intervention is promising.

Our results also showed lower BMI and decreased visceral fat in the intervention group, but not in the control group, although this was nonsignificant [*P* = 0.06 and *P* = 0.20, respectively, for 6 months changes from baseline (Table 3)]. Adiposity promotes breast cancer carcinogenesis by (i) stimulating chronic inflammation (6) and (ii) increasing concentrations of testosterone and estrogen (57), dysregulating leptin, adiponectin (7, 8), and insulin resistance (9). Low lean mass and/or increased BMI are predictors of poor prognosis in patients with breast cancer. For every 5 kg/m^2^ increase in BMI, a 7% increase in any second cancer and a 13% increase in obesity-related cancers has been reported (50). Sustained weight loss is associated with a lower risk of breast cancer, and the beneficial effects of exercise on disease-free survival are greater in overweight and obese women (58). Thus, lifestyle changes during radiotherapy may not only improve side effect profiles and enhance recovery from breast cancer treatments in overweight women but also improve breast cancer–specific outcomes. Furthermore, our results showed an increase in glucose and HbA1c levels only in control participants (Table 4). Metabolic syndrome is associated with a 73% increase in breast cancer mortality (59). Although adiponectin levels decreased in both groups, they tended to be nonstatistically higher in the intervention group than in the control at 6 months (Table 4). Higher preoperative adiponectin levels are associated with reduced breast cancer mortality by 61% (60). Together, diet and exercise interventions may improve breast cancer prognosis in patients by promoting beneficial adipose and metabolic profiles.

Most body composition parameters were decreased in the control and intervention groups, with no significant differences between the groups (Table 3). Breast cancer treatment leads to an increase in adipose tissue and accelerates the loss of lean mass (3), and exercise during and after treatment can oppose these effects (15). We found no differences in lean or fat mass at any of the timepoints between the groups, likely due to our sample size and short-term follow-up. Patients also typically started endocrine treatment following the completion of radiotherapy, which may confound these results. Surprisingly, we found a significant decrease in lean mass compared with baseline in the intervention group and a decrease in fat mass in the control group (Table 3). The decrease in fat mass likely contributed to the lower leptin levels observed in the control group over time (61). It is unclear whether these are a result of exercise intervention, dietary intervention, a combination of both, and/or a healthier lifestyle in the control group. The latter is less likely given that the intervention group had significantly more very active minutes during the baseline week versus controls (Fig. 2F). Alternatively, simply giving the control group a Fitbit, education on the benefits of diet/exercise, and encouraging them to use the Fitbit in the 1-week baseline period may have resulted in a lasting increase in activity levels and, thus, body composition measures. Moreover, studies have suggested that breast cancer survivors generally increase their fruit and vegetable consumption (62). Future studies that (i) objectively track activity levels in the entire control group with longer follow-up of participants, (ii) accurately track daily food and caloric intake as well as compliance with dietary recommendations, and (iii) randomize participants with consideration of baseline activity levels are needed to understand the impact of SOT on fat mass and lean mass.

The fat-free mass decreased in the intervention group compared with the baseline levels, but not in the control group (Table 3). Fat-free mass is metabolically active and is thus highly associated with RMR (63). Thus, these results are in line with the decrease in RMR in the intervention group at 6 months. This change could be reflective of general weight loss and/or lower protein intake in the intervention group compared with the control group which would lead to a reduction in lean mass. Alternatively, the loss of fat-free mass could be reflective of sarcopenia, which can predict severe toxicity following cancer treatment (64). Sarcopenia can be related to reduced food intake, increased energy expenditure, and elevated inflammation (65). The control group showed a decrease in anti-inflammatory IL1RA levels, and the intervention group showed a reduction in proinflammatory IL8 levels. Other studies have reported lower concentrations of proinflammatory cytokines in patients with breast cancer following regular exercise (18, 66). Given that our intervention was introduced during treatment, rather than after treatment as is commonly done, questions remain as to whether the timing of our study may have contributed to sarcopenia-resembling body changes. However, no signs of adverse outcomes were seen, and other studies show that exercise is safe and effective during active treatment (48, 49). The addition of endocrine treatment following radiotherapy may have also affected these results. Further analysis is necessary to understand whether diet modulation/intervention during treatment and/or the receipt of endocrine therapy might have resulted in the loss of fat-free mass over time in the intervention group.

The majority of participants completed the FFQ at all timepoints, showing improved nutrition at follow-up. Specifically, we observed reductions in GI and increases in ACS/AICR score in the intervention group at 3 months versus baseline. For individual component scores, greater adherence to dietary recommendations were seen in the intervention group at 3 months versus baseline across several components, which was also true in comparing changes in the intervention group with the control group at different times (Table 6). These data show promise in at least some areas of the nutritional component of the intervention. These changes may be due to the SOT intervention, likely the dietary counseling sessions and text messages, beyond what might be expected from survivorship education alone, given that there were significant differences when comparing the intervention arm with the control arm. Although, it should be noted that there were some improvements over time in the control group as well, with respect to avoiding added sugars and reducing GL, suggesting that the educational materials alone may have also had some beneficial effects on dietary habits. The value of diet in survivorship is highlighted by data suggesting improvement in overall survival among breast cancer survivors who adhere to healthy dietary habits (67). Unfortunately, the majority of breast cancer survivors fail to adhere to ACS dietary recommendations (68). Interventions such as SOT have the potential to improve this adherence. Some of the increased dietary adherence in the intervention group compared with the control group diminished at the 6-month timepoint, while increased intake of fruits and vegetables was maintained (Table 6). As such, some of the long-term benefits of these dietary changes may be limited without extension of the intervention. Of note, the control group showed lower GL during the trial period, which was unexpected, especially given that the GI was reduced overtime within the intervention group only. Although these differences were not significant with group*time interaction analyses, these findings may imply that the control group ate fewer, higher GI foods, at least transiently. While GL is important for cardiovascular disease, it has not been linked to cancer-related outcomes (69, 70).

This pilot study was limited by the small sample size, which lessened the statistical power of our analyses. As such, outside of some dietary improvements in the intervention group compared with the control group, all the other measures reported were within group changes with no significant differences between the groups over time. Nonetheless, the within group changes over time that occurred despite this small sample size are promising, and larger follow-up studies are warranted. The small sample size also prevented subanalysis of patients who adhered to the lifestyle interventions. Such analysis could be important in future studies, given the wide heterogeneity in participant activity levels throughout the study. The primary reason for patients not participating in the study was concern regarding time commitment/conflicts. The study design (three exercise and three dietary sessions along with text message reminders and Fitbit usage for motivation) was aimed at balancing time flexibility for participants with enough knowledge/guided sessions to encourage behavioral changes. Further optimizing interventions to take advantage of virtual environments and incorporating flexible scheduling may help increase patient participation in the future. Furthermore, a number of patients with data at enrollment failed to complete all follow-up scans, surveys, and questionnaires. In addition, our relatively short follow-up makes it unclear whether the benefits of SOT are sustained in the long term. Because FitBit use was only recommended to the intervention group, we were unable to objectively quantify physical activity changes using these data between control and intervention groups. In addition, although the control group was asked to stop using the Fitbit, they were allowed to keep the Fitbit during the study duration which may have still provided some motivation to stay active and as such confounded results. Indeed, 5 of 22 control group participants continued to wear the Fitbit for >70% of the days during the intervention period, with 4 of the 5 continuing to wear the Fitbit for at least 70% of the full 6-month tracking period. In total, however, our results show that SOT is feasible, beneficial, and well received in patients with breast cancer undergoing radiotherapy.

## Conclusion

Despite the known benefits of lifestyle interventions in improving QoL and decreasing CVD risk in patients with breast cancer undergoing adjuvant therapy, relatively few randomized controlled trials have assessed the combined effects of diet and exercise intervention in overweight women undergoing radiotherapy. Moreover, recent data suggest beneficial effects of lifestyle interventions during treatment in improving cardiovascular outcomes, a major cause of mortality in women with breast cancer. We piloted a 12-week lifestyle intervention starting from the time of whole-breast radiation in overweight women and showed that it was feasible with high rates of adherence. The intervention group had increased self-reported physical activity levels and QoL metrics, and select improved nutrition scores, while the control group had reduced QoL, anti-inflammatory markers, and increased markers of metabolic syndrome. While most of these improvements were within group effects, there were notable dietary improvements seen within the intervention group compared with the control group at different times. Larger-scale implementation of SOT with longer follow-up can shed light on the mechanistic factors that influence the impact of lifestyle interventions on QoL and breast cancer outcomes.

## Supplementary Material

Supplemental Tables 1-3 Supplementary Tables 1-3
