# Supplementary material for: Stay on Track: A Pilot Randomized Control Trial on the Feasibility of a Diet and Exercise Intervention in Patients with Breast Cancer Receiving Radiotherapy
Source: Cancer Res Commun. 2024 May 7;4(5):1211–26. doi: 10.1158/2767-9764.CRC-23-0148 (PMC11075661; doi:10.1158/2767-9764.CRC-23-0148)
Supplement: Supplemental Tables 1-3 — Supplementary Tables 1-3 [file crc-23-0148-s01.docx]

**Supplementary Tables:**

**Supplemental Table 1. Study Eligibility Criteria and Measures**

| **Eligibility criteria for participants** | |
| --- | --- |
| Inclusion: female ≥18 years, diagnosis of ductal carcinoma in situ (DCIS) or invasive breast cancer, planned whole breast RT following partial mastectomy without treatment of the level III axilla and supraclavicular lymph nodes, Karnofsky performance status ≥70, access to mobile phone with Bluetooth and texting capacity, understand and speak English, physically able to engage in intervention, BMI ≥ 25 or patient not meeting ACS guidelines for physical activity and diet (not engaging in at least 150 minutes of moderate intensity or 75 minutes of vigorous intensity activity each week or an equivalent combination, OR not consuming a healthy diet, with an emphasis on plant foods (like fruits and vegetables), OR not eating at least 2.5 cups of vegetables and fruits each day, OR not limiting consumption of processed and red meat, OR making an effort to choose whole grains instead of refined grain products, OR if they drink alcohol, they drink no more than 1 drink per day). If a patient with a BMI <25 answered “No” to any of the questions, they were eligible for the study.  Exclusion: distant metastatic disease, history of prior malignancy in the past 3 years other than non-melanoma skin cancer, prior radiotherapy to primary site or adjacent site that would result in overlapping radiation fields, serious orthopedic/cardiovascular/pulmonary/psychiatric/cognitive conditions. | |
| **Study Measures** | |
| **Feasibility** | Recruitment and retention rates |
| **Acceptability**  3 months | Satisfaction questionnaire |
| **Adherence**  Baseline, 3, 6 months | ***Godin Leisure PA****:* 4-items, time spent in light, moderate, strenuous activities over 7-days; reliability coefficient of 0.8 with VO_2_ measures.  ***Full Length NutritionQuest Block Questionnaire and Physical Activity Screening Tool***  ***Fitbit:*** Objective measure of physical activity. |
| **Patient**  **Reported**  **Outcomes**  Baseline, 3, 6 months | ***Piper Fatigue Scale*** *(PFS)*: 22 numerically scaled "0-10" items that measure 4 dimensions of subjective fatigue: behavioral/severity; affective meaning; sensory; and cognitive/mood. Sub-scale and total fatigue scores are calculated to provide reliable, valid assessments of fatigue in BC patients.^70^  ***FACT-B Questionnaire***: The patient reported outcomes measurement information systems a 27-item compilation of general questions divided into four primary QOL domains: Physical Well-Being, Social/Family Well-Being, Emotional Well-Being, and Functional Well-Being. |
| **Body Composition** **Outcomes**: Baseline, 3, 6 months | ***Body composition****:* DEXA imaging is a noninvasive, imaging technique that provides precise whole-body measurements of fat (total and visceral) and appendicular LST and takes 10 minutes to complete. Certified radiation technologists at MCW CTSI performed and analyzed using the DEXA scans by GE Lunar Medical Systems, Madison, Wisconsin; Software version 14.10.022) |
| **Biomarker Outcomes:** Baseline, 3, 6 months | *Biomarkers include:* ***Adipokines*** *(leptin, adiponectin);* ***Inflammation*** *(43 cytokine panel (Eve Technologies) including IL-6, TNF-α, CRP – as previously described in (71));* ***Insulin Resistance*** *(insulin, HbA1c, glucose).* |
| **Nutrition:**  Baseline, 3, 6 months | *Block 2014 Food Frequency Questionnaire (FFQ):* Full-length food frequency questionnaire for intake data over the past six- and one-month periods, based on National Health and Nutrition Examination Survey (NHANES) dietary recall data, offered by NutritionQuest. Includes demographic data, coding scheme for frequency questions and portion sizes for a wide variety of foods, beverages and supplements, food type questions, physical activity questions, and analysis output variables including diet, nutrient, and physical activity output variables. Includes a total of 440 questions with 1074 total output variables.  Individual component scores for ACS/AICR calculations were analyzed as follows: The fruits and vegetables component was calculated by summing cups of deep yellow-orange vegetables, tomatoes, dark green leafy vegetables, other vegetables, citrus melon berries, other solid fruit. These were converted to servings and servings >5, 3-5, 1-3, and <1 were scored as 3, 2, 1, 0 respectively. Whole Grains and Legumes/Soy was calculated by considering daily intake of total grains, daily intake of whole grains, servings of legumes, and servings of soy food. Patients consuming 50% or more of total grains as whole grains or 1 serving of legumes/soy were scored 3, consuming 40-50% of total grains as whole grains or 0.75-1 servings of legumes/soy were scored 2, consuming 30-40% of total grains as whole grains or 0.5-0.75 servings of legumes/soy were scored 1, and consuming <30% of total grains as whole grains and <0.5 servings of legumes/soy were scored 0. Limit red meat was calculated based on red and organ meat intake weekly, with patients consuming ≤18, <24, <30, and >30 ounces of red meat per week as 3, 2, 1, and 0 points respectively. Avoid processed meats was calculated by summing lunchmeats, hot dogs, and bacon (cured meats). Patients consuming ≤1, 1-2, 2-3, and 3 or more ounces of processed meat per day were scored 3, 2, 1, and 0 points respectively. Energy density was calculated by summing total solid, alcohol, and sweets to obtain total grams. Kilocalories per day, provided by the FFQ output, was then divided by total grams. Patients consuming <125, 125-175, 1175-225, and >225 calories per 100 grams were scored 3, 2, 1, and 0 points respectively. Avoid added sugars was calculated based on the FFQ output added sugar teaspoon equivalents. Patients consuming <6, 6.01–10, 10.01–14, and >14.0 teaspoons were scored 3, 2, 1, and 0 points, respectively. Alcoholic drinks were directly reported by the FFQ output. Patients consuming 0, 0-1, 1-2, and >2 drinks per day were scored 3, 2, 1, and 0 points, respectively. |

| **Supplementary Table 2. Representativeness of Study Participants** | |
| --- | --- |
| Cancer type(s)/subtype(s)/stage(s)/condition | Non-metastatic breast cancer |
| Considerations related to: | |
| Sex | Breast cancer is a predominantly female disease and is rare in men. Male breast cancer represents approximate 0.5 to 1% of all breast cancers diagnosed each year. |
| Age | The median age at the time of all breast cancer diagnoses is around 62. |
| Race/ethnicity | In the USA from 2015 to 2019, the overall breast cancer incidence rate was 127.8 cases per 100,000 among Black women and 133.7 cases per 100,000 among White women.   In Wisconsin, the incidence rate of breast cancer among White women is 136.7 and in Black women is 141.1, slightly higher than the national averages.   In Wisconsin, approximately 6.8% of the population is Black. This study has Black women making up 7.0% of participants. |
| Geography | In the US, about 287,850 new cases of invasive breast cancer will be diagnosed in 2022 and about 43,250 women die from breast cancer. In Wisconsin, around 5,460 new cases are estimated in 2023 with approximately 720 breast cancer deaths. |
| Other considerations | N/A |
| Overall representativeness of this study | All participants in this study were female.  The median age at the time of all breast cancer diagnoses is around 62, while in this study the median age was 57.  In Wisconsin, approximately 6.8% of the population is Black. This study has Black women making up 7.0% of participants.  The participants in the study were from the southeastern Wisconsin catchment area. |

**Supplemental Table 3. Study Satisfaction at 3 Months Among the Control and Intervention Group.** Responses for each category are shaded darkest grey for >50%, lighter grey for >25%, and lightest grey for >0%.

| **Intervention Group Satisfaction Survey (n=15)** | **Very Satisfied** | **Satisfied** | **Neutral** | **Dissatisfied or Very Dissatisfied** |
| --- | --- | --- | --- | --- |
| How satisfied were you with the Stay on Track intervention overall | **67%** | **33%** | **0%** | **0%** |
| How satisfied were you with the initial one-on-one introductory program review with the research coordinator that provided the intervention materials | **87%** | **13%** | **0%** | **0%** |
| How satisfied were you with the personal exercise sessions | **67%** | **27%** | **7%** | **0%** |
| How satisfied were you with the dietary counseling sessions | **73%** | **20%** | **7%** | **0%** |
| How satisfied were you with the weekly text messages | **33%** | **33%** | **27%** | **7%** |
| How satisfied were you with using the Fitbit personal activity tracker | **67%** | **33%** | **0%** | **0%** |
| How satisfied were you with the information binder you received | **60%** | **40%** | **0%** | **0%** |
|  | **Greatly Helped** | **Slightly Helped** | **Did not Help** | **Made Slightly/**  **Significantly Worse** |
| How did the stay on track intervention help you to improve your dietary habits to be in line with recommended guidelines during the 12wk study period | **47%** | **40%** | **7%** | **0%** |
| How did the stay on track intervention help you to change your dietary habits to be in line with recommended guidelines following the 12wk study period | **40%** | **53%** | **7%** | **0%** |
| How much do you think taking part in the stay on track intervention will help you to improve/continue your dietary habits to be in line with the recommended guidelines over the next year | **53%** | **40%** | **7%** | **0%** |
| How did the stay on track intervention help you to improve your activity habits to be in line with recommended guidelines during the 12wk study period | **47%** | **40%** | **13%** | **0%** |
| How did the stay on track intervention help you to change your activity habits to be in line with recommended guidelines following the 12wk study period | **33%** | **53%** | **13%** | **0%** |
| How will taking part in the stay on track intervention help you to improve and or continue your activity habits to be in line with recommended guidelines over the next year | **47%** | **47%** | **7%** | **0%** |
| How did the use of text messaging help you to improve your activity habits to be in line with recommended guidelines during the 12wk study period | **13%** | **47%** | **40%** | **0%** |
| How did the use of text messaging help you to improve your dietary habits to be in line with recommended guidelines during the 12wk study period | **20%** | **40%** | **40%** | **0%** |
| How did the use of the Fitbit activity monitor help you to change your activity habits to be in line with recommended guidelines following the 12wk study period | **67%** | **27%** | **7%** | **0%** |
| How did the use of the personal training sessions help you to change your activity habits to be in line with recommended guidelines following the 12wks study period | **27%** | **53%** | **20%** | **0%** |
| How did the use of dietary counseling sessions help you to improve your dietary habits to be in line with recommended guidelines during the 12wk study period | **53%** | **40%** | **7%** | **0%** |

| **Control Group Satisfaction Survey (n=15)** | **Very Satisfied** | **Satisfied** | **Neutral** | **Dissatisfied or Very Dissatisfied** |
| --- | --- | --- | --- | --- |
| How satisfied were you in taking part in the study overall | **47%** | **40%** | **7%** | **7%** |
|  | **Greatly Helped** | **Slightly Helped** | **Did not Help** | **Made Slightly/**  **Significantly Worse** |
| How did taking part in this trial help you to improve your dietary habits to be in line with recommended guidelines | **27%** | **47%** | **27%** | **0%** |
| How much do you think that taking part in trial will help you to improve/ continue your dietary habits to be in line with recommended guidelines over the next year | **53%** | **33%** | **13%** | **0%** |
| How did taking part in the trial help you to improve your activity habits to be in line with recommended guidelines | **33%** | **60%** | **7%** | **0%** |
| How will taking part in the trial help you to improve and or continue your activity habits to be in line with recommended guidelines over the next year | **60%** | **40%** | **0%** | **0%** |
